# Water Absorption Capacity Determines the Functionality of Vital Gluten Related to Specific Bread Volume

**DOI:** 10.3390/foods10020228

**Published:** 2021-01-23

**Authors:** Marina Schopf, Katharina Anne Scherf

**Affiliations:** 1 Leibniz-Institute for Food Systems Biology, Technical University of Munich, Lise-Meitner-Straße 34, 85354 Freising, Germany; m.schopf.leibniz-lsb@tum.de; 2 Department of Bioactive and Functional Food Chemistry, Institute for Applied Biosciences, Karlsruhe Institute of Technology (KIT), Adenauerring 20a, 76131 Karlsruhe, Germany

**Keywords:** baking quality, bread volume, gliadins, glutenins, vital gluten, water absorption, wheat

## Abstract

Vital gluten is often used in baking to supplement weak wheat flours and improve their baking quality. Even with the same recipe, variable final bread volumes are common, because the functionality differs between vital gluten samples also from the same manufacturer. To understand why, the protein composition of ten vital gluten samples was investigated as well as their performance in a microbaking test depending on the water content in the dough. The gluten content and composition as well the content of free thiols and disulfide bonds of the samples were similar and not related to the specific bread volumes obtained using two dough systems, one based on a baking mixture and one based on a weak wheat flour. Variations of water addition showed that an optimal specific volume of 1.74–2.38 mL/g (baking mixture) and 4.25–5.49 mL/g (weak wheat flour) was reached for each vital gluten sample depending on its specific water absorption capacity.

## 1. Introduction

Gluten is the most important by-product in the industrial production of wheat starch. During the process of washing out soluble wheat flour constituents and starch granules with water, gluten remains as a “rubber-like proteinaceous mass” [1]. After drying at mild temperatures below 55 °C, the final powdered product is called vital gluten. It regains its unique properties such as cohesiveness, elasticity, viscosity, and the ability to form films upon renewed addition of water [2]. Vital gluten mainly consists of proteins (about 73% to 80%) and a varying amount of lipids (5% to 8%) and carbohydrates (3% to 20%) [3]. Gluten proteins are storage proteins that are located in the starchy endosperm of wheat grains and can be separated into two fractions, the gliadins and the glutenins. Gliadins are predominantly monomeric proteins that are insoluble in salt solutions and water, but soluble in aqueous alcohols, e.g., 60% ethanol. Based on their relative molecular masses (M_r_) and similar amino acid sequences, gliadins are subdivided into ω5-, ω1,2-, α/β- and γ-gliadins. The M_r_ of ω5-gliadins is about 50,000 and that of ω1,2-gliadins about 40,000. For α-/β- and γ-gliadins, the M_r_ is similar and lies in the range of 28,000 to 35,000 [4]. Whereas ω-gliadins lack cysteine residues, α-gliadins have six and γ-gliadins eight cysteine residues that are responsible for the formation of intramolecular disulfide bonds [5]. The ability of glutenins to form intermolecular disulfide bonds leads to polymers with high M_r_ in the range of 80,000 to several millions [6]. A reducing agent, such as dithiothreitol (DTT) and increased temperatures above 60 °C are necessary to cleave disulfide bonds and release the monomeric high- (HMW-GS) and low-molecular-weight glutenin subunits (LMW-GS) that are soluble in aqueous alcohols, e.g., 50% propan-1-ol [7]. HMW-GS have a M_r_ of 67,000 to 88,000 and LMW-GS possess a M_r_ of 32,000 to 35,000 [8].

The dynamic interaction of gliadins and glutenins enables the formation of a viscoelastic gluten network when adding water to wheat flour or vital gluten powder [9]. Cuq, Abecassis, and Guilbert [10] described four different stages during the dough making and baking process. The first stage is the “water-flour mixing process”. During dough kneading, flour particles are hydrated after water addition, leading to increased mobility of their molecular chains, and swelling of the starch granules. Beside the impact of water, mechanical energy input during kneading leads to a redistribution of the flour particles and finally to the formation of a viscoelastic protein network (stage two). The mechanical energy causes a shearing effect and results in the destruction of discrete masses of gluten proteins [6]. By destroying disulfide bonds, large glutenin molecules can be extended beyond their equilibrium conformation [11]. A few hydrogen bonds still remain intact and ensure the elasticity of glutenins. During a resting period new disulfide bonds are formed resulting in a re-polymerization of glutenin macropolymer [12]. Gliadins are not directly involved in network formation, but act as plasticizers and increase entanglement spacing [11]. This leads to a higher viscosity and balances the elasticity of doughs [13]. The third stage is the heating process and the stabilization of the baked product. Starch undergoes gelatinization at temperatures above 60 °C, while the gluten network retains the gas generated by yeast during dough preparation and stabilizes the characteristic, foamy structure of wheat bread crumb. After cooling (stage four) the final product differs in water content, because the crumb has a high water content (35–40%), while the crust has a low water content (3–7%) [10].

Vital gluten is commonly used in the baking industry to supplement weak flours with low protein content and to improve their baking performance [14]. However, the functionality related to end-product quality differs batch-wise between vital gluten samples even from the same manufacturer. Understanding the functional properties of vital gluten is essential to expand its potential for use in various food and non-food products. Microscale extension and gluten aggregation tests were recently shown to be useful to predict the specific volumes of breads supplemented with vital gluten [15], but it is still not clear precisely how gluten functionality is determined by its composition, structure and chemical-physical properties especially in the complex dough system. One important factor which influences the viscoelastic properties of dough is the amount of added water. Water molecules act as plasticizers and have a softening effect on the dough structure [11], but the influence of water on the functionality of vital gluten used in, e.g., high-protein breads is largely unknown. Jekle and Becker [16] showed how different water concentrations influenced the network structure of gluten. The dough with below optimal water content showed a compact structure and the protein network was not evenly distributed. An optimal water content led to uniformly distributed and broadly oriented proteins, which were crosslinked by covalent, hydrophobic, ionic and hydrogen bonds. Doughs with above optimal water content had more aggregated proteins, which were less interconnected. Thus, the competition between starch and protein for available water plays a major role [17] and it is so far unknown, how the additional presence of vital gluten affects protein-starch-water interactions.

Based on the above we hypothesized that the amount of water influences the mobility of gliadins and glutenins during dough preparation and thus network development, which is dependent on the formation of intra- and intermolecular disulfide bonds and the hydration properties of starch and gluten. Therefore, the aim of our investigation was to study how the baking performance of ten vital gluten samples differing in functionality is influenced by the amount of water added to two different dough systems. To help explain the functional effects observed at the macroscopic scale, we complemented our analyses with the determination of the qualitative and quantitative protein composition and content of free thiols and disulfide bonds of the vital gluten samples.

## 2. Materials and Methods

### 2.1. Material

The ten vital gluten samples (G1–G10) tested in this study were obtained from five different suppliers (Crespel & Deiters GmbH & Co.KG, Ibbenbüren, Germany; Ireks GmbH, Kulmbach, Germany; Jäckering GmbH, Hamm, Germany; Kröner-Stärke GmbH, Ibbenbüren, Germany; Sigma Aldrich Chemie GmbH, Asten, Austria). Grains of the common wheat cultivar Elixer were kindly donated by Saaten Union GmbH (Moosburg, Germany). The wheat grains were milled into white flour with a Quadrumat Junior Mill (Brabender GmbH, Duisburg, Germany) and sieved (mesh size 200 µm). A baking mixture with wheat flour type 1050 (Walzmühle, Horb-Altheim, Germany), sunflower seeds (Walz-Mühle, Horb-Altheim, Germany), soy flour (Zimmermann, Neu-Ulm, Germany), sesame (Seeberger, Ulm, Germany), rye sourdough (Böcker GmbH & CoKG, Minden, Germany), lupine shots (Rapunzel Naturkost, Legau, Germany), linseeds (Walz-Mühle, Horb-Altheim, Germany), salt (Merck KGaA, Darmstadt, Germany) and roasted malt flour (Walz-Mühle, Horb-Altheim, Germany) was prepared. The yeast was provided by Brauerei Wieninger (Teisendorf, Germany). All chemicals were of analytical or higher quality and were purchased from Merck KGaA (Darmstadt, Germany), Sigma Aldrich (Steinheim, Germany) or SERVA Electrophoresis GmbH (Heidelberg, Germany). The reference material Prolamin Working Group (PWG)-gliadin [18] was donated by Prof. Dr. Koehler, Chairman of the Working Group for Prolamin Analysis and Toxicity. The NuPage Bis-Tris gel (10%) was obtained from Thermo Fisher Scientific Inc. (Braunschweig, Germany). The PageRuler unstained protein ladder was ordered from PanReac AppliChem (Darmstadt, Germany). Water for chromatographic separations was purified using a Milli-Q Gradient A10 system (Millipore, Schwalbach, Germany).

### 2.2. Analysis of Crude Protein Content

The crude protein content of the ten vital gluten samples G1–G10 was determined according to the method of Dumas using a FP-328 instrument (Leco Instrumente GmbH, Moenchengladbach, Germany). The crude protein content was calculated by multiplying the nitrogen content by a factor of 6.25 [19].

### 2.3. SDS-PAGE

SDS-PAGE was performed according to Lagrain et al. [20]. The XCell Surelock Mini-Cell electrophoresis system (Thermo Fisher Scientific, Braunschweig, Germany), NuPage Bis-Tris gels (10%) (Invitrogen, Carlsbad, CA, USA, pH 6.4, 1.0 mm × 10 wells) and the PageRuler Unstained Protein Ladder (Thermo Fisher Scientific, Braunschweig, Germany) as M_r_ marker were used. The vital gluten samples G1–G10 (4 mg) were dissolved in 1 mL of extraction buffer (293.3 mol/L sucrose, 246.4 mol/L Tris, 69.4 mmol/L SDS, 0.51 mmol/L EDTA, 0.22 mmol/L Coomassie Brilliant Blue R-250, 0.177 mmol/L phenol red, 0.105 mmol/L HCl, pH 8.5) for 12 h under reducing conditions (50 mmol/L DTT). Then, the samples were shaken at 60 °C for 10 min in a thermo shaker (MHR23, HLC BioTech, Bovenden, Germany) and centrifuged at 5000× *g* at 22 °C for 5 min (Microcentrifuge 1-15K, Sigma Laborzentrifugen GmbH, Osterode, Germany). For the electrophoresis 5 µL of the supernatant were used. Gel electrophoresis was performed with a 3-(*N*-morpholino)propanesulfonic acid (MOPS) buffer (50 mmol/L MOPS, 50 mmol/L Tris, 3.5 mmol/L SDS, 1 mmol/L EDTA, and pH 7.7) under reducing conditions (5 mmol/L DTT). The running conditions were as follows: current: 115 mA; voltage: 200 V, power: 30 W and time: 20–30 min. Proteins were fixed for 30 min with 12% trichloroacetic acid and stained with Coomassie Brilliant Blue R-250 for 30 min. The gel was then destained for 15 min with methanol/water/acetic acid (50/40/10, *v/v/v*) and then overnight with water/methanol/acetic acid (80/10/10, *v/v/v*).

### 2.4. Analysis of Vital Gluten Protein Composition by Sequential Extraction and RP-HPLC

The content of gluten proteins (gliadins and glutenins) was determined by modified Osborne fractionation and analytical reversed-phase high-performance liquid chromatography (RP-HPLC) [21]. To obtain the gliadins, 20 mg of vital gluten G1–G10 with added glass beads were extracted with 60% (*v/v*) aqueous ethanol (3 × 1.5 mL). The residue was then extracted with 50% (*v/v*) propan-1-ol, 0.05 mol/L Tris-HCl (pH 7.5), 2 mol/L (*w/v*) urea and 1% (*w/v*) DTT (3 × 1.5 mL, 60 °C, nitrogen atmosphere) to dissolve the glutenins. The extraction procedure involves vortex mixing for 2 min, then magnetic stirring for 10 min at 20–22 °C (gliadins) or stirring for 30 min at 60 °C (glutenins), followed by centrifugation for 25 min at 4600× *g* at 22 °C (Multifuge X3 FR, Heraeus, Hanau, Germany). Appropriate extracts were combined and made up to a volume of 5.0 mL with the corresponding extraction solution. For RP-HPLC analysis of the fractions, a Hitachi Merck instrument (VWR, Darmstadt, Germany) with a Dionex Acclaim 300 C18 column (3 µm, 30 nm, 2.1 × 150 mm, Thermo Fisher Scientific, Braunschweig, Germany) and the software LaChrom Elite was used. The instrument settings were: column temperature; 60 °C, UV detection; 210 nm, flow rate; 0.3 mL/min, injection volume; 20 µL, solvents; water/trifluoroacetic acid (TFA) (999/1, *v/v*) (A) and acetonitrile/TFA (999/1, *v/v*) (B). The solvent gradient was 0 min; 24% B, 20 min; 56% B, 21 min; 90% B, 26 min; 90% B, 27 min; 24% B, 37 min; 24% B. PWG-gliadin (11.6 to 46.6 µg, dissolved in 60% (*v/v*) ethanol) was used for the calibration and the calculation of protein contents [18]. All extractions were performed in triplicates.

### 2.5. Determination of Free Thiols and Disulfide Bonds

The content of free thiols and disulfide bonds was determined photometrically using Ellman’s reagent. All measurements were performed in triplicates. To obtain free thiols, the vital gluten samples G1-G10 (10 mg) were first dissolved with 900 µL of buffer solution (0.05 mol/L Na_2_HPO_4_/NaH_2_PO_4_, pH 6.5, 3.0 mol/L urea, 0.001 mol/L EDTA, and 2.0% SDS, *w/v*). The samples were shaken for 60 min at 500 rpm and 22 °C (MHR23, HLC BioTech, Bovenden, Germany). Then, 100 µL of a 0.1% 5,5’-Disulfanediylbis(2-nitrobenzoic acid) (DTNB) (*w/v*) solution in buffer were added and the mixture was incubated for 45 min at 500 rpm and 22 °C. Finally, the samples were centrifuged for 5 min at 11,000× *g* at 22 °C (Multifuge X3 FR, Heraeus, Hanau, Germany) and the absorbance of the supernatant was measured with the spectrophotometer UV-2401PC (Shimadzu, Kyoto, Japan) at 412 nm. Reduced glutathione was used for calibration and conversion of the absorbance values into the content of free thiols (µmol SH/g protein). To quantitate disulfide bonds, the vital gluten samples G1–G10 (2 mg) were reduced with 200 µL of NaBH_4_ (2.5%, *w/v*) and shaken for 60 min at 500 rpm and 50 °C (MHR23, HLC BioTech, Bovenden, Germany). Then, 100 µL of HCl (1 mol/L) were added to each sample and they were shaken for 30 min at 500 rpm and 22 °C. Then the samples were treated in the same way as for the determination of free thiols. Oxidized glutathione was used for calibration and conversion of the absorbance values into the content of disulfide bonds (µmol SS/g protein).

### 2.6. Microbaking Tests

All microbaking tests were performed in triplicates under controlled conditions (temperature 22 ± 2 °C, relative humidity ≥60%). Two recipes were used for the microbaking experiments. Recipe 1 was adapted from a typical high-protein bread recipe and contained 7.5 g of a baking mixture (composition: 8 g linseeds, 5 g sunflower seeds, 10 g soy flour, 1.1 g salt, 10 g lupine shots, 4 g wheat flour type 1050, 3 g rye sourdough, 2 g sesame and 0.5 g roasted malt flour), 2.5 g vital gluten and 0.25 g yeast. Recipe 2 consisted of 7.5 g of weak wheat flour (cultivar Elixer), 2.5 g vital gluten, 0.25 g yeast, 0.1 g coconut fat, 0.2 g salt and 0.1 g sugar. For both recipes, all ingredients were first kneaded to the respective optimum (22 °C, 550 BU, [22]), followed by adding different amounts of water in a farinograph-E (Brabender GmbH, Duisburg, Germany) (Table 1). The dough ball was formed manually and left to rest for 20 min at 30 °C in a water-saturated atmosphere. This was followed by the final proofing step (30 °C, 40 min) and baking (185 °C increasing to 255 °C, 10 min) on a fully automated baking line [23]. After a cooling time of 2 h, the volume was determined using a laser-based scanning device (VolScan Profiler, Stable Micro Systems, Godalming, UK). To compensate for, e.g., dough losses during dough preparation, the specific volume was calculated by dividing the bread volume by the final dough weight.

### 2.7. Statistical Analysis

Significant differences between vital gluten samples were determined by one-way analysis of variance (ANOVA) with Tukey’s test (*p* ≤ 0.05) using SigmaPlot 12.0 (Systat Software, San José, CA, USA). Spearman’s correlation coefficients (r) to determine the correlations between all quantitative parameters, as well as the coefficient of variation for the contents of gluten protein types were calculated in Origin 2019 (OriginLab Corporation, Northampton, MA, USA).

## 3. Results

### 3.1. Qualitative Protein Distribution

**Figure 1:**
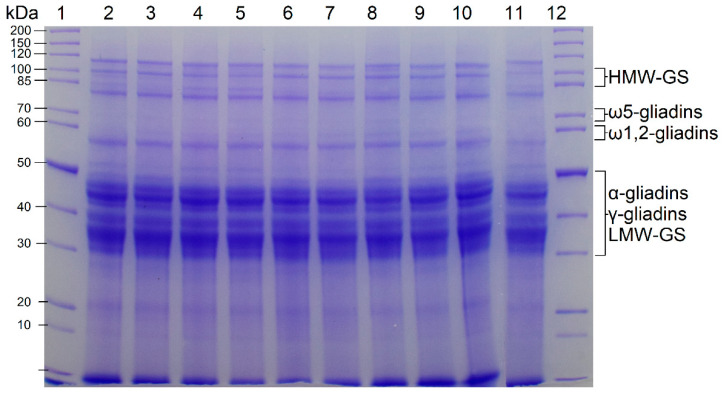
Bis-Tris SDS-PAGE (10%) of protein extracts from vital gluten samples G1–G10. Lanes 1 and 12 show the marker, the other lanes show the band patterns of vital gluten samples G1–G10. Lane 2: G1; Lane 3: G2; Lane 4: G3; Lane 5: G4; Lane 6: G5; Lane 7: G6; Lane 8: G7; Lane 9: G8; Lane 10: G9 and Lane 11: G10.

The SDS-PAGE patterns of the vital gluten samples G1–G10 (reducing conditions) are shown in Figure 1. The bands corresponded well to the expected band patterns for wheat gluten proteins with the typical bands for HMW-GS, ω5-gliadins, ω1,2-gliadins, as well as α- and γ-gliadins and LMW-GS [5]. The band patterns of all vital gluten samples showed great similarity, indicating that the qualitative composition was comparable with only minor variations in the intensity of some bands.

### 3.2. Qualitative and Quantitative Protein Composition

The absolute content of ω5- ωb-, ω1,2-, α- and γ-gliadins, HMW-GS and LMW-GS are shown in Supplementary Table S1. The total gluten content was between 752.0 mg/g (G2) and 844.5 mg/g (G9). Compared to the crude protein content, which ranged from 817.2 mg/g (G7) to 862.2 mg/g (G1), the recovery rate for the sum of gluten proteins determined by RP-HPLC was between 90.3% (G2) and 100.0% (G4). The gliadin-to-glutenin ratio ranged from 1.5 (G5) to 2.7 (G3). The content of gluten protein types between vital gluten samples was similar, but there were some significant differences (Table S1). The highest variability occurred in the content of α-gliadins and LMW-GS. The values ranged from 243.4 mg/g (G2) to 315.5 mg/g (G3) for α-gliadins and from 152.2 mg/g (G3) to 230.7 mg/g (G9) for LMW-GS. Vital gluten samples G3 and G4 showed higher values for the gliadin types and the resulting total gliadin content compared to the other vital gluten samples. Furthermore, their glutenin content was lower and reached values of 224.9 mg/g (G3) and 229.3 mg/g (G4). This led to higher gliadin-to-glutenin ratios of 2.7 (G3) and 2.6 (G4). In contrast, sample G9 had a high glutenin content of 343.8 mg/g and sample G2 had a low gliadin content of 471.2 mg/g. This resulted in low gliadin-to-glutenin ratios of 1.7 (G2) and 1.5 (G9).

**Figure 2:**
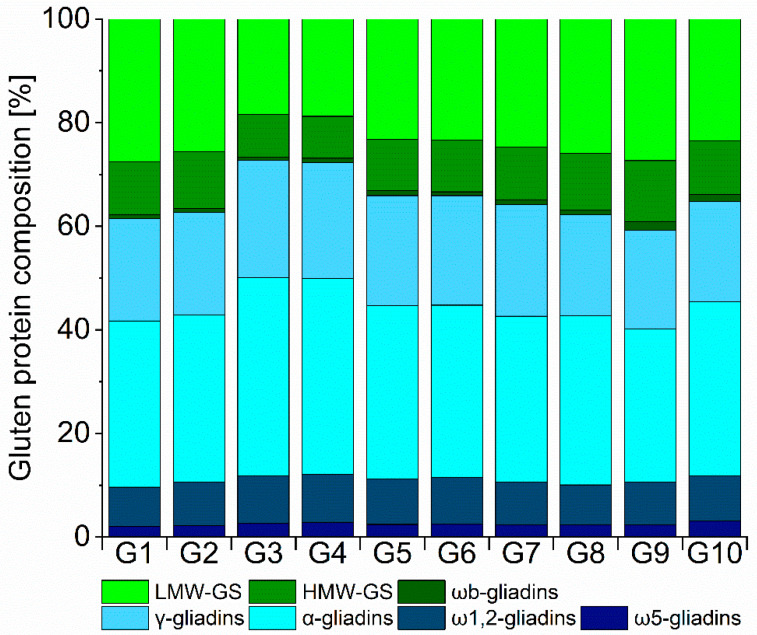
Relative protein composition of vital gluten samples G1–G10 (%): Distribution of vital gluten samples into ω5-, ω1,2-, α- and γ-gliadins as well as ωb-gliadins, high molecular weight glutenin subunits (HMW-GS) and low-molecular-weight-glutenin subunits (LMW-GS).

As expected. considering the absolute content, the protein composition relative to the sum of extractable proteins showed similarities between the vital gluten samples (Figure 2). Samples G1 and G2, G3 and G4, G5 and G6 as well as G9 and G10 were from the same supplier, respectively. Especially samples from the same supplier showed great resemblance in their distribution. The relative amounts were 2.1–3.1% for ω5-gliadins, 0.6–1.6% for ωb-gliadins, 7.5–9.2% for ω1,2-gliadins, 29.6–38.2% for α-gliadins and 19.1–22.7% for γ-gliadins. For glutenins, the relative amounts were 8.1–11.9% for HMW-GS and 18.4–27.5% for LMW-GS. The coefficient of variation over all ten samples was used to assess overall variability between the samples and it was 0.1 for all gluten protein types, except ωb-gliadins (0.3). In line with the SDS-PAGE results, we found only small variations in the qualitative and quantitative protein composition of the ten vital gluten samples studied.

### 3.3. Content of Free Thiols and Disulfide Bonds

The content of free thiols ranged from 3.2 µmol SH/g protein (G8) to 6.5 µmol SH/g protein (G4) while the content of disulfide bonds was between 43.2 µmol SS/g protein (G4) and 61.1 µmol SS/g protein (G2). There were some significant differences between the vital gluten samples G1–G10 (Table S2). The coefficient of variation considering all ten vital gluten samples was 0.2 (free thiols) and 0.1 (disulfide bonds), again pointing to comparatively small overall variability between the samples.

### 3.4. Microbaking Tests

Microbaking tests were performed on a scale of 10 g using two different recipes. Both recipes contained 25% vital gluten (G1–G10), which resulted from preliminary experiments with 20%, 25% and 30% vital gluten. The final selection was made with 25% vital gluten addition, because this content showed the best results in crumb formation in terms of gas bubble homogeneity. The optimal kneading time for both dough systems can be seen in Table 1. Based on the hypothesis that the mobility of the gluten proteins has a major impact on the formation of the gluten network, a baking series with different water contents was performed for each vital gluten sample. The overall highest specific volumes and their corresponding water amounts, respectively, are shown in Table 1.

#### 3.4.1. Microbaking Tests Using the Baking Mixture

**Figure 3:**
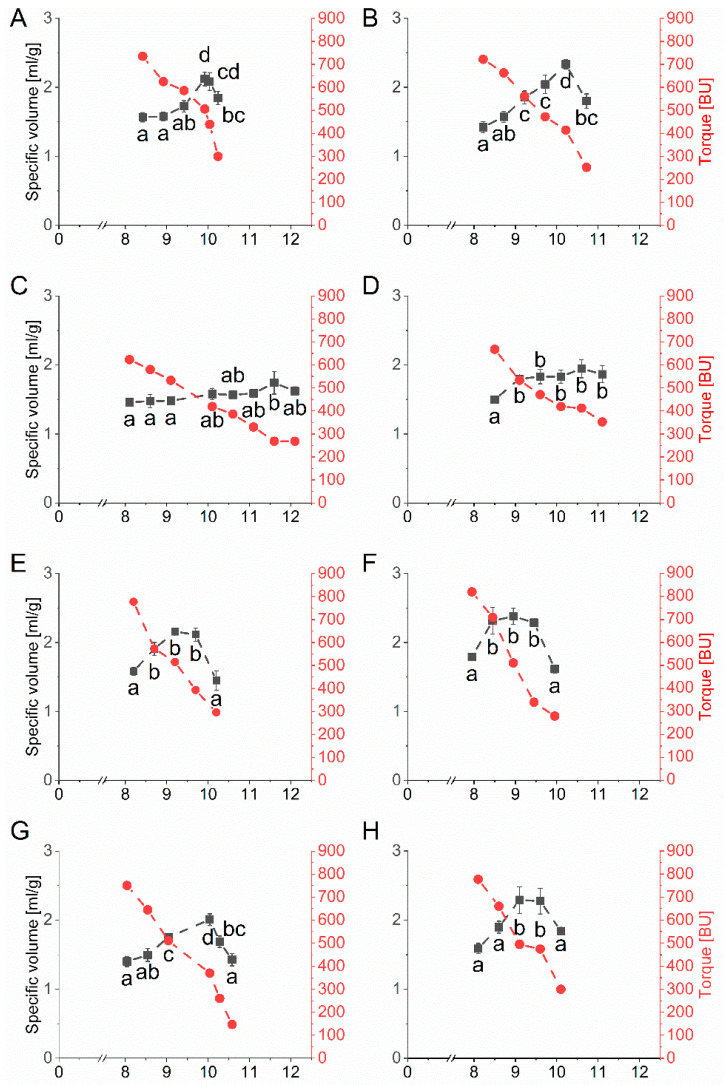

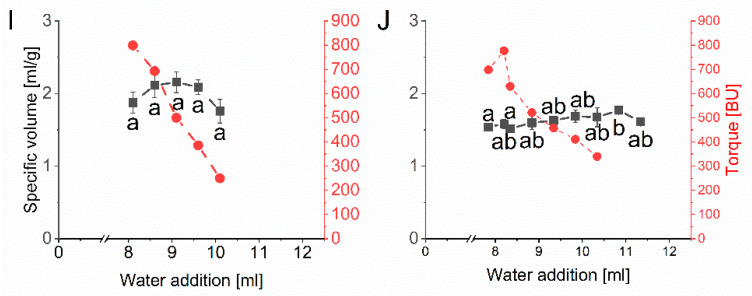
Mean values for specific volume (mL/g) and torque measured in Brabender units (BU) depending on the amount of water (mL) determined by microbaking tests with the baking mixture (10 g scale, *n* = 3) and 25% of each vital gluten sample: (**A**): G1; (**B**): G2; (**C**): G3; (**D**): G4; (**E**): G5; (**F**): G6; (**G**): G7; (**H**): G8; (**I**): G9; (**J**): G10. Significant differences are indicated by different small letters (one-way ANOVA, Tukey’s test, *p* ≤ 0.05); relative standard deviations of triplicate experiments were below 10%.

The specific volume and the corresponding torque as a function of water addition to the baking mixture containing one vital gluten sample, respectively, can be seen in Figure 3. All ten vital gluten samples G1–G10 reached an optimal specific volume with an individual amount of water. The specific volume was between 1.74 mL/g (G3) and 2.38 mL/g (G6). The overall coefficient of variation considering the specific maximal volumes of all ten vital gluten samples was only 0.1, with an overall mean of 2.09 mL/g. The water amount needed to reach the optimal specific volume was different between vital gluten samples and ranged from 8.95 mL (G6) to 11.60 mL (G3) (Table 1).

#### 3.4.2. Microbaking Tests Using Weak Wheat Flour

**Figure 4:**
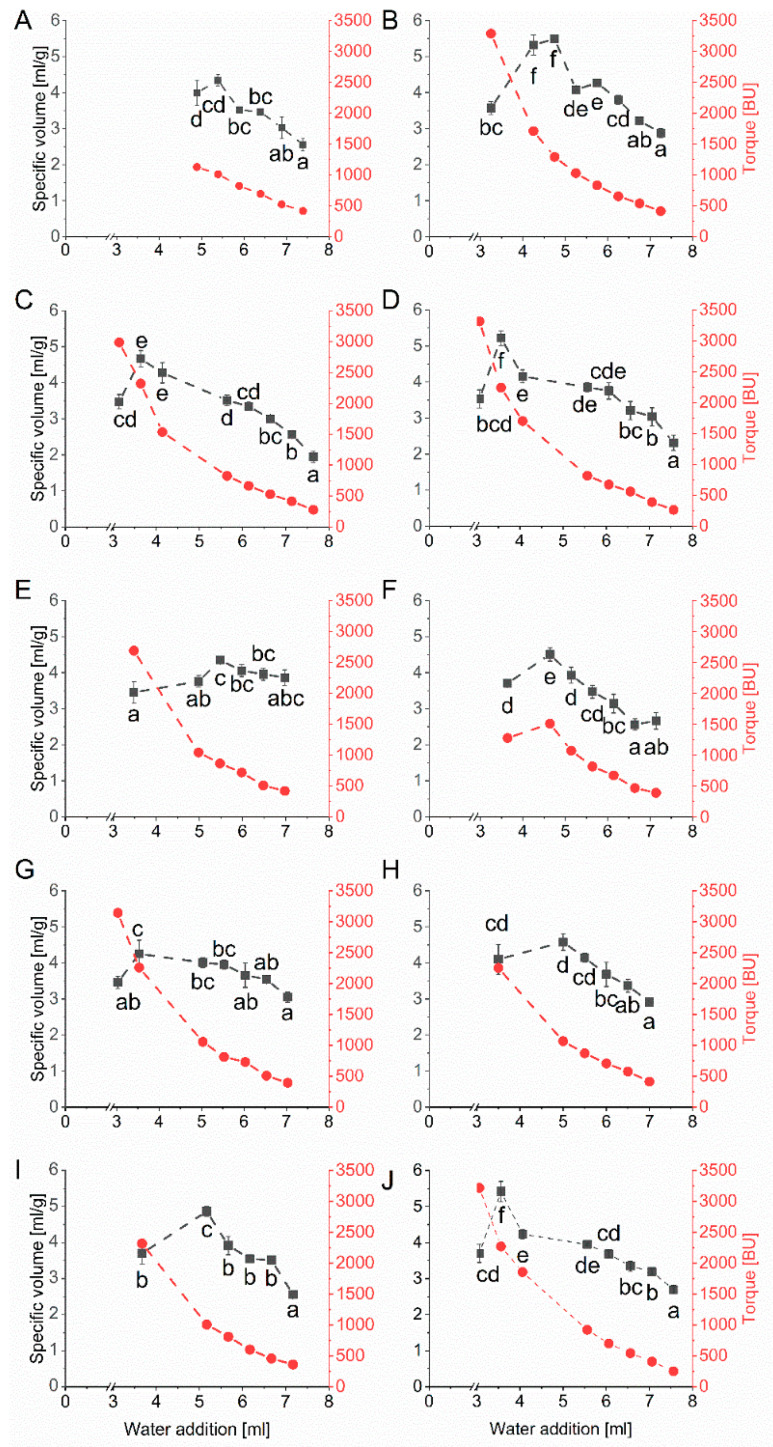
Mean values for specific volume (mL/g) and torque measured in Brabender units (BU) depending on the amount of water [mL] determined by microbaking tests with weak wheat flour (10 g scale, *n* = 3) and 25% of each vital gluten sample: (**A**): G1; (**B**): G2; (**C**): G3; (**D**): G4; (**E**): G5; (**F**): G6; (**G**): G7; (**H**): G8; (**I**): G9; (**J**): G10. Significant differences are indicated by different small letters (one-way ANOVA, Tukey’s test, *p* ≤ 0.05); relative standard deviations of triplicate experiments were below 10%.

The second recipe based on a weak wheat flour (cultivar Elixer) was used to exclude a potential bias of the results using the baking mixture with its rather complex composition. Wheat cultivar Elixer belongs to quality class C of the German wheat classification system (i.e., wheat intended for feed) and had a low protein content of 8.7% (based on dry matter). The specific volumes and their corresponding kneading times were determined depending on the amount of water added (Figure 4). Similar to the results of the microbaking test with the baking mixture, the specific volume reached an optimum at an individual amount of water. The specific volumes were between 4.25 mL/g (G7) and 5.49 mL/g (G2). The overall coefficient of variation was 0.1 considering the maximal specific volumes of all ten vital gluten samples, with an overall mean of 4.77 mL/g. The amount of water, which was necessary to achieve the optimal specific volume was between 3.53 mL (G7) and 5.48 mL (G5) (Table 1).

## 4. Discussion

Both dough systems showed optima in the specific bread volume for each vital gluten sample at a specific water content, but the maximal specific volumes of both recipes were not correlated with each other (r = −0.05, *p* = 0.87). The main difference between both recipes was the presence of oilseeds in the baking mixture. The oilseeds increased the complexity of the dough matrix and changed the dynamics of water absorption, because gluten molecules had to compete not only with starch, but also with the oilseeds, for the available water. To develop an optimal gluten network, doughs made from the baking mixture thus required more water than those made from weak wheat flour, which is in line with earlier reports using a multi-grain mix [24]. Additionally, breads of weak wheat flour had higher specific volumes compared to the baking mixture. One possible reason was the difference in complexity of the dough matrices. In weak wheat flour, mainly starch and gluten molecules were part of the gluten network. In contrast, the baking mixture had additional ingredients such as oilseeds, which are likely to affect the interaction of gluten molecules to a greater degree than shown recently for, e.g., wheat bran [25]. It is reasonable to assume that gluten polymers had a wider distance to each other, which limited their interconnection so that only restricted formation of covalent bonds may take place. This led to a weaker gluten network and therefore a reduced gas holding capacity, which resulted in lower specific volumes.

**Figure 5:**
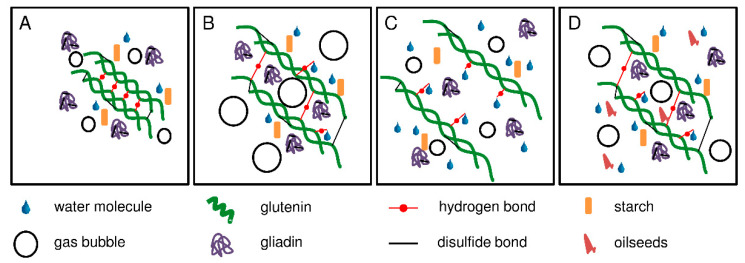
Schematic representation of gluten network formation depending on the water content. (**A**): weak wheat flour with below optimal water content; (**B**): weak wheat flour with optimal water content; (**C**): weak wheat flour with above optimal water content and (**D**): baking mixture with optimal water content.

In general, the dough system can be divided into three different phases: the gas phase, the protein network, and the continuous phase of free water with starch granules and water-soluble components [26]. The protein network is responsible for the viscoelastic behavior and determines the functional properties of the dough. However, the precise molecular structures are not yet fully clear, but there are various models to explain gluten network formation. All models suggest that glutenin polymers are responsible for the network formation, whereas gliadins are not directly involved. Gliadins increase the viscosity of the dough by weakening the interactions between glutenin chains [13]. Graveland et al. [27] proposed a model, where the backbone is composed of HMW-GS only, but with lateral attachments of LMW-GS. A more recent model by Lindsay and Skerritt [28] agreed with this model, but added branches containing LMW-GS and HMW-GS. The strength of the gluten network is determined by several factors, e.g., crosslinking of gluten polymers by disulfide bonds, hydrogen bonds, ionic bonds, van der Waals forces and water addition. Our study showed that different amounts of water had a significant impact on the specific bread volume. The mobility of gluten molecules is limited in doughs with below optimal water content and, therefore, the glutenin subunits are not able to interconnect properly. The limited mobility could be due to the existing hydrogen bonds between glutenin molecules. In the presence of water, some protein-protein hydrogen bonds are replaced by water-protein hydrogen bonds [29]. The lack of hydration maintains protein-protein hydrogen bonds and thus leads to dense masses preventing gliadins from being embedded in the network structure. The consequence is a weak viscoelastic protein network, which cannot retain the gas developed during dough making (Figure 5A).

In contrast, doughs with above optimal water content have a high mobility of gluten molecules. A possible explanation could be a high amount of water-protein hydrogen bonds that lead to the formation of a less interconnected gluten network (Figure 5C). These doughs suffer from softening of the structure, which involves a lower extensibility and thus a reduced gas holding capacity compared to doughs with optimal water content [30]. The consequence are breads with a small specific volume.

Doughs with the optimal amount of water develop a strong viscoelastic gluten network, resulting in high specific volumes (Figure 5B,D). The optimal specific volume was reached at a different amount of water for each gluten sample. When using the optimized kneading and water addition parameters, the overall variability of specific volumes between the gluten samples was low with a coefficient of variation of 0.1 (Table 1). In turn, this means that there is no “weak” vital gluten as long as the water quantity is optimal. Within one dough system only the vital gluten samples were responsible for the differences. Due to their overall high degree of resemblance in terms of protein composition, as also reported earlier [31], and content of free thiols and disulfide bonds, we conclude that the disparities regarding baking performance when using a standardized amount of water was induced by different water absorption capacities.

This conclusion is supported by calculating Spearman’s correlations between the specific volumes of both recipes, all quantitative protein composition parameters (Table S1) and the content of free thiols and disulfide bonds (Table S2). The highest correlation coefficients were r = −0.58 (*p* = 0.07) for α-gliadins considering the baking mixture and r = −0.38 (*p* = 0.27) for γ-gliadins considering the weak wheat flour, but neither were significant, as reported earlier when considering a set of 46 vital gluten samples [13]. The content of free thiols was also not related to the specific volumes achieved with the baking mixture (r = −0.42, *p* = 0.23) or the weak wheat flour (r = 0.52, *p* = 0.12). The same was true for the content of disulfide bonds (r = 0.33, *p* = 0.35, baking mixture and r = 0.30, *p* = 0.40, weak wheat flour). This means that the different functionality of the vital gluten samples in terms of baking performance and gluten network formation could not be explained by differences in protein composition or available thiols/disulfide bonds.

Water absorption is determined by the hydrophilicity or hydrophobicity of gluten proteins. Previous studies showed that the addition of gluten or glutenin to flour increased water absorption [32]. The “free” water that is not absorbed by the gluten-starch matrix determines the consistency of the dough structure. Taking one example for the baking mixture, vital gluten G1 had a glutenin content of 305.7 mg/g and reached its optimal specific volume with a water addition of 9.92 mL. In comparison, vital gluten G6 showed a glutenin content of 266.8 mg/g and had the optimal specific volume at a water addition of only 8.95 mL, confirming that lower glutenin contents contribute to lower water absorption.

## 5. Conclusions

The protein composition of vital gluten samples G1–G10, especially from the same supplier, was similar, because RP-HPLC, SDS-PAGE and thiol/disulfide analyses showed only small differences. Both dough systems resulted in an optimal specific volume for each vital gluten sample depending on the amount of water added. This means that each vital gluten could reach a high specific volume with an adapted water addition to take the individual water absorption capacities into account. However, in routine baking procedures this is not done, because it is time-consuming and costly, so that large variations in gluten functionalities occur using the same water addition for all samples. While this may be close to the optimum for one vital gluten sample by chance, it may be far from it for a second gluten sample. In this case, gluten network formation during kneading will be almost optimal for sample one, but far from optimal for sample two, simply because of different water absorption capacities. Further analysis should consider studying the specific protein-protein interactions depending on the water content on a molecular level, e.g., the formation of disulfide bonds during the kneading process.

## Figures and Tables

**Table 1:** Optimal kneading times determined by farinograph-E of the baking mixture and the weak wheat flour with 25% of the vital gluten samples G1–G10 as well as optimal specific volumes (mL/g) (*n* = 3) and corresponding water amounts (mL). Significant differences are indicated by different small letters (one-way ANOVA, Tukey’s test, *p* ≤ 0.05).

Vital Gluten	Baking Mixture			Weak Wheat Flour		
	Kneading Time	Specific Volume ^1^	Water Amount	Kneading Time	Specific Volume ^1^	Water Amount
	(min)	(mL/g)	(mL)	(min)	(mL/g)	(mL)
G1	36	2.12 ^b,c,d,e^	9.92	15	4.34 ^a,b^	5.39
G2	35	2.33 ^d,e^	10.22	16	5.49 ^d^	4.75
G3	26	1.74 ^a^	11.60	12	4.66 ^a,b,c^	3.64
G4	32	1.95 ^a,b,c^	10.60	11	5.22 ^c,d^	3.56
G5	39	2.15 ^c,d,e^	9.20	23	4.34 ^a,b^	5.48
G6	33	2.38 ^e^	8.95	20	4.51 ^a,b^	4.64
G7	39	2.00 ^a,b,c,d^	10.04	21	4.25 ^a^	3.53
G8	39	2.29 ^d,e^	9.10	22	4.58 ^a,b,c^	5.00
G9	38	2.16 ^c,d,e^	9.10	25	4.87 ^b,c,d^	5.17
G10	28	1.77 ^a,b^	10.84	9	5.42 ^d^	3.56

^1^ Relative standard deviations of triplicate experiments were below 10%.

## Supplementary Materials

The following are available online at https://www.mdpi.com/2304-8158/10/2/228/s1, Table S1: Comparison of ten vital gluten samples G1–G10 based on absolute contents (mg/g) of different gluten protein types ω5-, ωb-, ω1,2-, α- and γ-gliadins, high-molecular-weight glutenin subunits (HMW-GS) and low-molecular-weight glutenin subunits (LMW-GS), the gliadin-to-glutenin ratio (glia/glut) and the crude protein content. Table S2: Contents of free thiols (µmol SH/g protein) and disulfide bonds (µmol SS/g protein) of ten vital gluten samples G1–G10.
