# Supplementary material for: Water Absorption Capacity Determines the Functionality of Vital Gluten Related to Specific Bread Volume
Source: Foods. 2021 Jan 23;10(2):228. doi: 10.3390/foods10020228 (PMC7910979; doi:10.3390/foods10020228)
Supplement: Supplementary file 1 [file foods-10-00228-s001.pdf]

foods MDPI

Supplementary Material

Water Absorption Capacity Determines the Functionality of Vital Gluten Related to Specific Bread Volume

Marina Schopf, Katharina Anne Scherf

Table S1. Comparison of ten vital gluten samples G1-G10 based on absolute contents [mg/g] of different gluten protein types ω5-, ωb-, ω1,2-, α- and γ-gliadins, high-molecular-weight glutenin subunits (HMW-GS) and low-molecular-weight glutenin subunits (LMW-GS), the gliadin to glutenin ratio (glia/glut) and the crude protein content. The values are given as means (n = 3) that had less than 10 % relative standard deviation. Different small superscript letters indicate significant differences between the samples within each column (one-way ANOVA, Tukey's test, $p < 0.05$).

vital gluten	ω5-gliadins	ω1,2-gliadins	α-gliadins	γ-gliadins	total gliadins	ωb-gliadins	HMW-GS	LMW-GS	total glutenins	gluten	glia/glut	crude protein
	[mg/g]	[mg/g]	[mg/g]	[mg/g]	[mg/g]	[mg/g]	[mg/g]	[mg/g]	[mg/g]	[mg/g]		[mg/g]
G1	16.7^{a}	59.7^{a}	254.9^{a,b}	156.7^{a}	488.0^{a,b}	5.6^{a}	81.7^{b}	218.4^{c,d}	305.6^{b,c}	793.6^{a,b}	1.6^{a,b}	862.2^{e}
G2	17.0^{a,b}	62.4^{a,b}	243.4^{a}	148.4^{a}	471.2^{a}	5.6^{a}	82.7^{b}	192.6^{b,c}	280.9^{b}	752.0^{a}	1.7^{a,b}	832.5^{a,b,c,d}
G3	21.6^{b,c}	76.2^{c}	315.5^{c}	186.9^{d}	600.1^{d}	5.1^{a}	67.6^{a}	152.2^{a}	224.9^{a}	825.0^{a,b}	2.7^{c}	837.7^{c,d}
G4	23.5^{c}	75.6^{c}	307.7^{c}	181.8^{c,d}	588.6^{c,d}	6.6^{a,b}	67.0^{a}	155.7^{a}	229.3^{a}	817.9^{a,b}	2.6^{c}	818.1^{a,b}
G5	20.0^{a,b,c}	72.8^{b,c}	277.3^{b}	175.8^{b,c,d}	546.0^{b,c}	8.1^{a,b}	82.0^{b}	192.1^{b,c}	282.2^{b}	828.2^{a,b}	1.9^{b}	842.5^{c,d}
G6	20.0^{a,b,c}	70.3^{a,b,c}	260.3^{a,b}	165.7^{a,b,c}	516.3^{a,b}	5.7^{a}	78.4^{a,b}	182.7^{a,b}	266.8^{a,b}	783.1^{a,b}	1.9^{b}	834.0^{b,c,d}
G7	18.9^{a,b}	64.4^{a,b,c}	252.0^{a,b}	169.6^{a,b,c,d}	504.8^{a,b}	7.2^{a,b}	80.3^{b}	194.1^{b,c}	281.5^{b}	786.3^{a,b}	1.8^{a,b}	817.2^{a}
G8	18.5^{a,b}	60.9^{a}	257.0^{a,b}	153.0^{a}	489.3^{a,b}	6.9^{a,b}	86.3^{b}	203.5^{b,c,d}	296.8^{b}	786.1^{a,b}	1.6^{a,b}	828.1^{a,b,c}
G9	19.8^{a,b,c}	69.7^{a,b,c}	249.9^{a,b}	161.3^{a,b}	500.6^{a,b}	13.0^{c}	100.1^{c}	230.7^{d}	343.8^{c}	844.5^{b}	1.5^{a}	846.2^{d}
G10	24.0^{c}	68.2^{a,b,c}	260.7^{a,b}	150.5^{a}	503.4^{a,b}	10.2^{b,c}	81.2^{b}	182.6^{a,b}	274.0^{b}	777.3^{a}	1.8^{b}	831.0^{a,b,c,d}

foods

MDPI

Table S2. Contents of free thiols [μmol SH/g protein] and disulfide bonds [μmol SS/g protein] of ten vital gluten samples G1-G10. The values are given as means (n = 3) that had less than 10% relative standard deviation (RSD). Different small superscript letters indicate significant differences between the samples within each column (one-way ANOVA, Tukey's test, $p < 0.05$).

Vital gluten	Free thiols	RSD	Disulfide bonds	RSD
	[μmol SH/g protein]	[%]	[μmol SS/g protein]	[%]
G1	$4.2^{b,c}$	5.6	$54.4^{b,c,d}$	6.6
G2	$5.6^{d,e}$	8.5	61.1^{d}	7.7
G3	$5.8^{e,f}$	7.2	$47.9^{a,b,c}$	1.0
G4	6.5^{f}	4.0	43.2^{a}	4.3
G5	$4.0^{a,b,c}$	6.1	$45.7^{a,b}$	5.6
G6	$4.8^{c,d}$	8.3	$51.2^{a,b,c}$	8.9
G7	$3.8^{a,b}$	7.8	$48.5^{a,b,c}$	4.9
G8	3.2^{a}	6.6	$47.6^{a,b,c}$	4.2
G9	$3.4^{a,b}$	4.7	$55.8^{c,d}$	5.1
G10	$5.1^{d,e}$	6.7	$52.1^{a,b,c}$	5.8
